# Performance of an artificial intelligence-powered smartphone application in the UK clinical settings: ECG automation compared to healthcare professionals

**DOI:** 10.1186/s43044-025-00689-1

**Published:** 2025-10-16

**Authors:** Ahmed Kassem, John Folkes, Sahil Mukherjee, James Rosengarten

**Affiliations:** 1 Cardiology Department, East Kent Hospitals University NHS Foundation Trust, Ashford, United Kingdom; 2 King’s College London, London, United Kingdom

## Abstract

**Background:**

The electrocardiogram (ECG) is widely used in clinical practice, but accurate interpretation requires significant expertise. Variability in training leads to inconsistent diagnostic accuracy amongst healthcare professionals. Artificial intelligence (AI) applications, such as PMCardio (Powerful Medical, Samorin, Slovakia), can digitise and interpret ECGs. While validated in selected populations, its performance compared to clinicians in UK practice has not been assessed.

**Methods:**

Seventy-six healthcare professionals interpreted eight ECG traces (seven abnormal, one normal). Their performance was compared with the PMCardio application. Accuracy and time were recorded.

**Results:**

Healthcare professionals achieved a mean accuracy rate of 67.1% (SD 24.0%), improving with seniority (junior 60%, mid-level 67.5%, senior 80%). PMCardio achieved perfect accuracy on the tested ECGs. Clinicians interpreted faster (median 23.7 s, range 9.1 s) compared to PMCardio (39.0 s, range 8.0 s), noting that the application’s timing included operational steps such as loading and capturing ECG images.

**Conclusions:**

PMCardio demonstrated higher diagnostic accuracy than healthcare professionals but required longer interpretation times. Given the small dataset (8 ECGs) and lack of patient context, results should be interpreted cautiously. While AI tools may support clinicians and enhance consistency, they may also introduce uncertainty for less experienced users. Further studies with larger, real-world datasets are needed before widespread adoption.

## Background

Cardiovascular disease (CVD) represents one of the leading causes of death [1]. Within CVD, there has been a steady rise in machine learning use (2012–2020) with exponential growth in 2021 [2]. Importantly, deep learning is superseding machine learning [3]. The use of Artificial Intelligence (AI) focuses on heart arrhythmia and CVD; this is unsurprising since Atrial Fibrillation (AF) represent 40% of heart-related problems in young people (18–35 years) and Coronary Arterial Disease (CAD) affects 60% of the elderly population (65 years and above) [4].

Recently, AI has also shown extensive use in investigations in cardiology: Electrocardiograms (ECG), echocardiography, coronary angiography, computed tomography, and cardiac magnetic resonance imaging [5]. Since most CVD patients are asymptomatic or have unclear symptoms, AI can provide cheap and flexible support to cardiologists [6]. AI can increase diagnostic throughput [7] and accuracy by reducing error rates, especially for high-risk conditions, as well as variability and bias [8]. Also, it can assist in speeding up diagnosis time, enabling earlier diagnosis [9], hence allowing for timely interventions to be implemented [4], which is critical to prevent irreversible damage and also important in allowing for personalised treatment [4].

Within diagnostics, the ECG is traditionally dependent on manual analysis by cardiologists and is prone to subjective bias and inter-observer variability [10]. It is further impacted by a lack of standardised teaching of ECG interpretation [11]. Since an ECG gathers standardised, reproducible physiological data stored in a consistent, compact digital format, it is ideal for AI implementation [12]. Hybrid models have been proposed for ECG data analysis, and AlexNet, ResNet, and GoogLeNet are the most commonly used. [3].

PMCardio (Powerful Medical, Samorin, Slovakia) is a CE-marked mobile application capable of digitising an ECG recording and producing a clinical diagnosis. Validation studies have explored its use in specific chest pain populations, but the performance compared to different clinicians has not been evaluated in the UK. This study aims to evaluate the practical value of a leading AI ECG interpretation application by comparing its accuracy and speed to that of practicing healthcare professionals in different disciplines with varying levels of expertise and experience.

## Methods

The objectives of this study were twofold: (1) to compare the mean accuracy and speed of ECG interpretation by a leading AI application with that of non-cardiologist clinicians across varying levels of seniority, and (2) to conduct a preliminary real-world assessment of the application's performance in both acute and non-acute clinical settings. [11].

This was a prospective, observational diagnostic accuracy study conducted at a District General Hospital. The study comprised 76 healthcare professionals (general practice physicians, doctors, and paramedics), asked to interpret seven high-risk ECG traces and one normal ECG trace. This data was then compared to interpretations by the AI mobile application PMCardio. The accuracy and time taken for each interpretation were recorded. The healthcare professionals understood their role in the analysis and consented to involvement. The sample ECG traces were randomly selected from an online ECG bank [13] and included Normal Sinus Rhythm, anterior ST-segment Elevation Myocardial Infarction (STEMI), posterior STEMI, Left Main Coronary artery occlusion STEMI, Left Bundle Branch Block (LBBB), Complete Heart Block, Atrial Flutter, and Atrial Fibrillation. The ECG traces were simultaneously run on the PMCardio mobile application.

PMCardio is a mobile application that can be downloaded on any smart mobile device. The user captures a digital image of any 12-lead ECG acquired through standard clinical practice. The app can handle all common layout formats. The application then processes the ECG trace and provides a result with multiple diagnoses, with the most likely diagnosis classified as “high confidence”. The application could also give additional clinical guidance for management if further patient information is provided, such as age, gender, and presenting complaint.

Participants were informed that the research aimed to compare the accuracy of a mobile AI application to healthcare professionals in interpreting ECG traces. Demographic information collected from participants included: the healthcare profession, department, and level of seniority. No additional clinical information was provided to assist with interpretation. Participants were isolated during the survey to ensure unbiased responses, and each response was timed using a stopwatch.

A total of 76 healthcare professionals participated; 88% were doctors and 12% were paramedics. Amongst them, 73% worked in Internal Medicine, 7% in the Emergency Department, 8% as General Practitioners. 34% were junior-level (foundation doctors or senior house officers), 42% were mid-level (specialty registrars), and 24% were senior-level (consultants). For paramedics, seniority was self-identified due to the lack of standardised levels.

The study was conducted over a 3-month duration from 01/03/2024 to 01/06/2024 and was done at a District General Hospital in the UK.

## Results

Table 1 presents the diagnostic accuracy of ECG interpretations by healthcare professionals compared to the PMcardio mobile application, showing the average percentage of correct diagnoses for each.

**Table 1:** Presents the diagnostic accuracy of ECG interpretations by healthcare professionals compared to the PMcardio mobile application, showing the average percentage of correct diagnoses for each.

	Total		
	Healthcare professional	AI	P-value
Normal sinus rhythm	67 (88.2%)	76 (100%)	0.004*
Anterior STEMI	70 (92.1%)	76 (100%)	0.03*
Posterior STEMI	46 (60.5%)	76 (100%)	<0.001*
Left bundle branch block	39 (51.3%)	76 (100%)	<0.001*
Left main coronary artery occlusion/ STEMI	29 (38.2%)	76 (100%)	<0.001*
Complete heart block	48 (63.2%)	76 (100%)	<0.001*
Atrial flutter	42 (55.3%)	76 (100%)	<0.001*
Atrial fibrillation	65 (85.5%)	76 (100%)	0.001*

(Table 1) *statistically significant at *p*-value < 0.05. This was done using the McNemar test. Data was analysed using IBM SPSS for Windows (Version 26.0).

When comparing the diagnosis time of various ECG types by AI and professionals, AI consistently took a longer time across all ECG types. For example, AI took 35 (4.5) seconds to diagnose NSR compared to 24.66 (17.10) seconds for professionals. Similarly, AI diagnosis of Anterior STEMI took 37 (5.6) seconds while professionals took 21.88 (17.77) seconds. The trend continued with AI at 35 (5.2) seconds for Posterior STEMI versus 22.82 (17.15) seconds for professionals, and 41 (4.2) seconds for LBBB compared to 22.14 (20.36) seconds for professionals. For more critical conditions like left main coronary artery occlusion/STEMI, AI took 43 (5.1) seconds while professionals took 28.86 (22.33) seconds. CHB was diagnosed by AI in 40 (4.6) seconds versus 26.91 (23.19) seconds by professionals. Atrial Flutter and Atrial Fibrillation also followed this pattern, as AI took 38 (5.4) and 40 (3.5) seconds, respectively, compared to 22.72 (16.37) and 19.75 (17.05) seconds by professionals. This was done using the Kruskal-Wallis test (3 groups) and the Mann-Whitney U (2 groups). The significance level was set at *p*-value <0.05. Data were analysed using IBM SPSS for Windows (Version 26.0).

We conducted our study based on the following hypotheses: Null Hypothesis (H0); the mean accuracy rate of healthcare professionals is equal to the AI application's accuracy rate. Alternative Hypothesis (H1); the mean accuracy rate of healthcare professionals is different from the AI application's accuracy rate.

Descriptive statistics for the accuracy rates of healthcare professionals yielded a mean accuracy rate of 67.1% (standard deviation: 24.0%). Accuracy improved with seniority: junior 60%, mid-level 67.5%, and senior-level 80%, whereas the AI mobile application, PMCardio, was 100% accurate across the 8 test ECGs.

Speed of interpretation was higher amongst healthcare professionals (median 23.7 s, range 9.1 s) compared to PMCardio (39.0 seconds, range 8.0 seconds), which included time to load the application, capture and format the ECG, and process the image.

Using NumPy/SciPy/Pandas Python software package (version 3.10), a one-sample t-test compared the mean accuracy rate of healthcare professionals with the AI application’s perfect accuracy rate (100%), resulting in a value of -11.965 and a *p*-value of 4.575366^-19^. Significance was judged at the 0.05 level, allowing us to reject the null hypothesis. The confidence interval assumes that identical and independent experiments are being used, while the 8 ECG traces were not identical in their ease of interpretation.

## Discussion

This study suggests that the use of an AI-powered ECG mobile application improves the accuracy rate of ECG interpretation within a clinical setting. Implications of AI in medicine, however, are multifaceted. In respect to the use of the PMCardio mobile application in a clinical setting, this application can prove to be helpful to support healthcare professionals in their interpretation, especially when they are not fully confident in their ECG interpretation skills. Given that there is no formal training technique on ECG interpretation, there remains to be a variance in expertise between healthcare professionals of different specialties when it comes to interpreting an ECG, with healthcare professionals more exposed to ECGs at a higher level of expertise. However, given the small number in each subgroup within this study’s cohort, the results of the accuracy of interpretation amongst different seniority levels and professional backgrounds should be interpreted cautiously.

This application can also provide a platform for the self-development of healthcare professionals, as it can be used in a non-clinical setting to allow them to test themselves on different ECGs and relay whether they were able to come up with the right diagnosis, which this application can provide. The time for diagnosis is longer with the use of the AI application, taking into account the time needed to use the device application in terms of logging into the mobile phone, and taking a clear picture of the ECG trace and does not distinguish between pure algorithm processing time and user operation time. The PMCardio application also presents multiple diagnoses/interpretations with the most likely one tagged as ‘high significance’, and others tagged with varying degrees of significance, which might confuse less experienced healthcare professionals and could lead to unnecessary activations of primary percutaneous intervention centres if acute myocardial infarction is incorrectly suggested as a lower probability interpretation. Hence, further research and audits would be required locally within hospitals and also on a national level to allow for PMCardio to be recommended for use as a standard for ECG diagnosis.

Patients may show less tolerance for AI-made errors compared to errors made by clinicians [14], and alongside clinicians share concerns of medical AI’s potential to reduce human interactions and patients’ trust in healthcare communication [15].

Data concerns involving patients’ privacy and security breaches (susceptibility to hacking and data access from private 3rd parties) [16], and violations of informed consent [17]. AI’s ability to predict demographic details makes complete de-identification difficult. Therefore, regulations are needed to ensure AI systems are safe, effective, and ethical in use [18], with challenges regarding monetisation [12] and intellectual property [19], however, regulations may not guarantee that algorithms will generate fair decisions [18]. Even for studies to report results, correct guidelines are still necessary [2]. An important aspect for consideration in using AI in clinical practice is adequately educating the medical workforce [20] in implementing AI in their practice.

Expanding further into AI, and its black box & white box algorithms; “Black Box” is where ML and DL lack explainability (where users cannot understand the reasoning behind each prediction) [21]. “White box” algorithms like logistic regression and decision trees are more transparent [22], with clarity of how data inputs are used by AI models to produce outputs [20]. This lack of transparency may negatively impact human oversight, error detection and propagate health care biases [23]. Some studies have combined deep features with traditional features of the models to address this issue [3], and this enhancement of interpretability without compromising quality can improve widespread implementation in healthcare.

Moreover, to add to the complexity of application of AI in healthcare practice, there are several considerations regarding imbalanced data, such as the variation of ethnicities, different cardiac conditions, and different ECG types, and there is a lack of standardised data [2], impacting AI-generated predictions [16] and outcomes [18]. Therefore, adaptable AI models that can act across ECG datasets should be encouraged. Otherwise, deficient model prediction may lead to over-detection of previously undiagnosed conditions, leading to increased use of diagnostic tests and treatments that may be unnecessary or harmful [24].

AI implementation could encourage collaboration between healthcare professionals and data management, e.g. The Data Science Institute has supported the use of AI implementation [20]. Collaboration could enable standardised integration to streamline existing clinical workflows [20], avoiding information overload and overwhelming healthcare systems with false positive results [12].

There is also an opportunity to scale to low and middle-income nations; expansion to wearable biosensor technologies or devices to encourage continuous patient monitoring, personalised care and early diagnosis within reach of larger populations [25]. With time, it could also be scaled to non-cardiac diagnoses.

In summary, black box and data imbalance awareness should shape future technology development and are crucial to increasing the effectiveness of the use of AI in healthcare [26]. Also, the current trend in adequate education of healthcare professionals and their acceptance would potentially lead to an expanded use of AI in their practice.

## Study limitations

The small sample size of healthcare professionals (and corresponding subcategories of different backgrounds) and the number of ECG traces used could limit the strength of the study. The method of ECG interpretation was not in line with real-life clinical practice, given that no clinical information was provided to the healthcare professionals alongside the ECG traces. Finally, the use of ECG traces from an online ECG bank, which could have already been used in the ML of the application’s coding, may have contributed to inflating the accuracy rate of the PMCardio mobile application.

## Conclusions

This study shows that, when compared to healthcare professionals, the AI mobile application, PMCardio, has a high accuracy rate in diagnosing ECG traces, although interpretation time is longer, noting that the application’s timing included operational steps such as loading and capturing ECG images. Nevertheless, this study supports previous findings that computerised ECG interpretation enhances the accuracy, confidence, and performance of healthcare professionals, emphasising the importance of integrating AI into clinical practice [27][27, 28]. The utilisation of AI to modernise healthcare settings requires further research. Adopting these tools will likely have a high economic burden on the short-term, given extra funding would be required for paying subscriptions to new smartphone applications as the one discussed in this study, as well as, funding for research to ensure patient safety and validation of efficiency, especially in state-funded services such as the NHS (National Health Service) in the UK, but they potentially offer improvements in patient care on the long-term.

Future studies should aim to validate PMCardio across larger, real-world, and more diverse patient datasets to strengthen the evidence base for its clinical utility. Importantly, successful adoption within clinical practice will require integration of this smartphone application into existing clinical workflows. This could be in the form of a decision-support tool for healthcare professionals working in the frontlines, hence assisting with rapid triage, and providing standardised interpretation to reduce inter-observer variability, while ensuring that final clinical judgement remains with healthcare professionals.

## Data Availability

No datasets were generated or analysed during the current study.

## Abbreviations

SD: Standard deviation
AI: Artificial intelligence
ML: Machine learning
DL: Deep learning
CVD: Cardiovascular disease
AF: Atrial fibrillation
CAD: Coronary arterial disease
ECG: Electrocardiogram
STEMI: ST-segment elevation myocardial infarction
LBBB: Left bundle branch block
CHB: Complete heart block
NSR: Normal sinus rhythm
CE: Conformité européenne (European Conformity)
NHS: National health service
